# Modelling Co-Infection of the Cystic Fibrosis Lung by *Pseudomonas aeruginosa* and *Burkholderia cenocepacia* Reveals Influences on Biofilm Formation and Host Response

**DOI:** 10.1371/journal.pone.0052330

**Published:** 2012-12-21

**Authors:** Alessandra Bragonzi, Ilaria Farulla, Moira Paroni, Kate B. Twomey, Luisa Pirone, Nicola Ivan Lorè, Irene Bianconi, Claudia Dalmastri, Robert P. Ryan, Annamaria Bevivino

**Affiliations:** 1 Division of Immunology, Transplantation and Infectious Diseases, Infections and Cystic Fibrosis Unit, San Raffaele Scientific Institute, Milan, Italy; 2 Technical Unit for Sustainable Development and Innovation of Agro-Industrial System, ENEA Casaccia Research Centre, Rome, Italy; 3 Department of Microbiology, BioSciences Institute, University College Cork, Cork, Ireland; University of Technology Sydney, Australia

## Abstract

The Gram-negative bacteria *Pseudomonas aeruginosa* and *Burkholderia cenocepacia* are opportunistic human pathogens that are responsible for severe nosocomial infections in immunocompromised patients and those suffering from cystic fibrosis (CF). These two bacteria have been shown to form biofilms in the airways of CF patients that make such infections more difficult to treat. Only recently have scientists begun to appreciate the complicated interplay between microorganisms during polymicrobial infection of the CF airway and the implications they may have for disease prognosis and response to therapy.

To gain insight into the possible role that interaction between strains of *P. aeruginosa* and *B. cenocepacia* may play during infection, we characterised co-inoculations of *in vivo* and *in vitro* infection models. Co-inoculations were examined in an *in vitro* biofilm model and in a murine model of chronic infection. Assessment of biofilm formation showed that *B. cenocepacia* positively influenced *P. aeruginosa* biofilm development by increasing biomass. Interestingly, co-infection experiments in the mouse model revealed that *P. aeruginosa* did not change its ability to establish chronic infection in the presence of *B. cenocepacia* but co-infection did appear to increase host inflammatory response.

Taken together, these results indicate that the co-infection of *P. aeruginosa* and *B. cenocepacia* leads to increased biofilm formation and increased host inflammatory response in the mouse model of chronic infection. These observations suggest that alteration of bacterial behavior due to interspecies interactions may be important for disease progression and persistent infection.

## Introduction

Chronic airway infections cause a progressive deterioration of lung tissue, a decline in pulmonary function and, ultimately, respiratory failure and death in cystic fibrosis (CF) patients [1]. CF airways are often colonized by opportunistic bacterial pathogens with *Pseudomonas aeruginosa* being one of the most regularly isolated organisms [2], [3]. Other frequently isolated opportunistic bacterial pathogens include *Haemophilus influenzae*, *Staphylococcus aureus*, *Stenotrophomonas maltophilia* and *Burkholderia cenocepacia* [4], [5], a species included in the *Burkholderia cepacia* complex (Bcc).

Recently, molecular approaches for community profiling have revealed that CF airways harbor far more organisms that evade detection by routine cultivation than originally thought [6]–[8]. It has been suggested that the bacterial community composition may be a better predictor of disease progression than the presence of stand alone opportunistic pathogens [9]. With this enhanced insight into the bacterial community composition of the CF airway researchers have begun to investigate the interspecies interactions that occur within these diverse polymicrobial infections and to examine the impact they may have on the disease progression and host response.

In this study, we have focused our attention on *P. aeruginosa* and *B. cenocepacia*, two important opportunistic pathogens that are rarely eradicated by antibiotic therapy and contribute significantly to the disease progression [10]. It was believed for sometime that the incidence of co-infection was low in most patients, with the occasional occurrence of super-infection of *B. cenocepacia* setting in on preexisting chronic *P. aeruginosa* infection leading to a rapid downturn in patient prognosis [11]–[13]. However, with the recent publication of numerous CF airway microbiome studies this position is being re-evaluated [14]–[16]. Indeed, the ecological interactions between these two bacterial species as well as the complex interplay between them and the host immune system during co-infections of the CF lung remains to be fully understood.

Here we examined the complex interactions between strains of *P. aeruginosa* and *B. cenocepacia* throughout growth in batch cultures, during attachment to plastic, in biofilm formation and in a mouse model of chronic infection with the aim to understand the impact of co-infecting bacteria on pathogenesis and bacterial physiology. A clear dominant negative effect of *P. aeruginosa* over planktonically grown *B. cenocepacia* was found, while a synergistic interaction between the two species takes place in biofilm formation *in vitro* leading to increased *P. aeruginosa* biomass. *In vivo* results demonstrated that the capacity of *B. cenocepacia* to establish long-term chronic infection was strongly damped by the presence of *P. aeruginosa* in both wild-type and CF mice. Nevertheless, *B. cenocepacia* altered the host inflammatory response in dual-species interaction. These observations suggest that co-infection of *B. cenocepacia* may facilitate *P. aeruginosa* persistence by interfering with host innate defense mechanisms.

## Results

### Competition between clinical and environmental pairs of *P. aeruginosa* and *B. cenocepacia* strains in planktonic co-cultures

**Figure 1:**
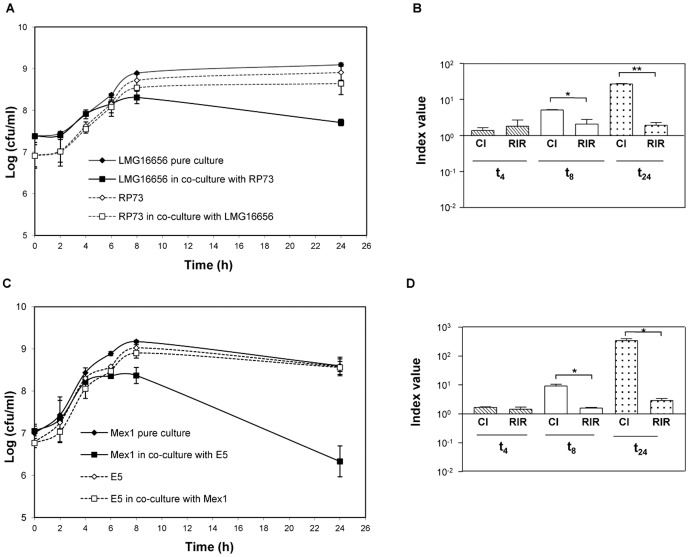
Single and dual species batch growth curves and competitive index values. The two species were individually cultured or co-cultured at a 1∶1 ratio and grown for 24 h in NB medium at 37°C with vigorous aeration. Colony-forming unit counts (CFU) were determined at 0, 2, 4, 6, 8 and 24 h of bacterial growth. The results are the mean of Log (CFU ml^−1^) values of three separated assays. Key: (A) Growth of clinical *P. aeruginosa* RP73 and *B. cenocepacia* LMG16656 strains in single and dual cultures; (B) Competitive index (CI) and relative increase ratio (RIR) generated from single and dual cultures of clinical *P. aeruginosa* RP73 and *B. cenocepacia* LMG16656 strains; (C) Growth of environmental *P. aeruginosa* E5 and *B. cenocepacia* Mex1 strains in single and dual cultures; (D) Competitive index (CI) and relative increase ratio (RIR) generated from single and dual cultures of environmental *P. aeruginosa* E5 and *B. cenocepacia* Mex1 strains. CI and RIR were calculated as described in *Materials and Methods*. Each value represents the mean of RIR and CI values from three separate assays, and the bars indicate standard deviations. * = *P*<0.05, ** = *P*<0.01 in the Student's t test.

The clinical and environmental pairs of *B. cenocepacia* and *P. aeruginosa* strains were investigated in planktonic co-cultures at five points during the growth curve (2, 4, 6, 8 and 24 hours). First, the respective paired strains had a similar generation time in pure culture (RP73 *versus* LMG16656 *P* = 0.128; E5 *versus* Mex1 *P* = 0.683) demonstrating similar bacterial growth during the early exponential phase. The analysis of the population dynamics of both paired clinical and environmental bacterial strains revealed that the *B. cenocepacia* growth significantly decreased from 8 h up to 24 h in the presence of *P. aeruginosa* (*P*<0.05) (Figure 1A, C), whilst *P. aeruginosa* growth was not affected by the presence of *B. cenocepacia* (*P*>0.05).

For a clearer comprehension of the differences in growth between *P. aeruginosa* and *B. cenocepacia* in single *versus* mixed cultures, we calculated the Competitive Index (CI) and a CI-like index, the Relative Increase Ratio (RIR). As shown in Figure 1 (B, D), the CI of *P. aeruginosa* versus *B. cenocepacia* increased significantly until 24 h of bacterial growth and became significantly higher than the RIR at 8 h of bacterial growth (*P*>0.05), suggesting a clear dominant negative effect of *P. aeruginosa* over *B. cenocepacia* growth in late-exponential and stationary-phase liquid culture, irrespective of strains origin. Similar findings were obtained for the interaction of clinical *B. cenocepacia* LMG16656 strain with the laboratory *P. aeruginosa* PAO1 strain (Figure S1).

Next, we explored the effect of secreted compounds produced by *P. aeruginosa* on the planktonic growth of *B. cenocepacia*, and vice versa. Supernatants of *P. aeruginosa* caused the inhibition of *B. cenocepacia* grown in planktonic conditions, whereas secreted compounds from *B. cenocepacia* did not affect *P. aeruginosa* in all strain pairs tested (Figure S2). These results were not due to an increase in nutrient levels, since the addition of concentrated unused growth medium to cultures of *B. cenocepacia* did not affect their growth (*P*>0.05) (Figure S2). Taken together, these data demonstrate that *P. aeruginosa* as well as its extracellular products can inhibit *B. cenocepacia* growth in planktonic cultures.

### *P. aeruginosa* and *B. cenocepacia* interaction during attachment to microtiter plates

**Figure 2:**
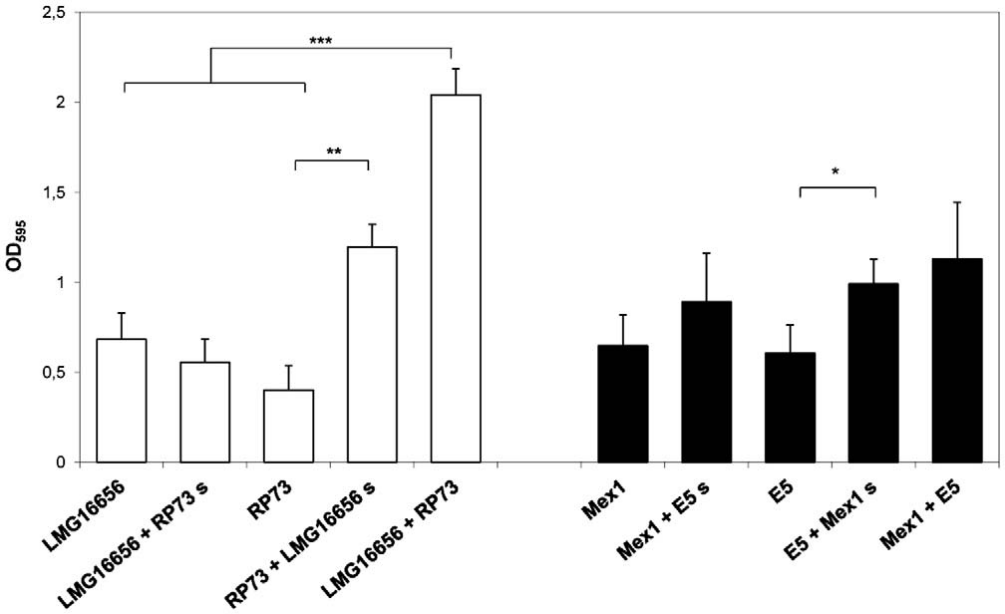
Biofilm formation by *P. aeruginosa* and *B. cenocepacia* strains in single and dual cultures. Bacteria were grown overnight in 96-well polyvinyl chloride flat-bottomed microtiter plates in NB medium at 37°C either individually cultured or co-cultured at a 1∶1 ratio or when individually cultured supplemented with sterile concentrated supernatant of the second organism at a final concentration of 1×. Biofilm biomass was quantified by staining with crystal violet and absorbance measurements at OD _595_. The values are means of three separated assays, and the bars indicate standard deviation. * = *P*<0.05, ** = *P*<0.01, *** = *P*<0.001 in Student's t test. S = supernatant.

Here, we quantified attached biomass of paired clinical and environmental *P. aeruginosa* and *B. cenocepacia* in single and mixed cultures in microtiter plates using the crystal violet (CV) assay. Results revealed that mixed colonies formed by the clinical *B. cenocepacia* LMG16656 and *P. aeruginosa* RP73 strains increased in mass only at 24 h of growth if compared to their pure culture counterparts (*P*<0.01) (Figure 2). Very similar results were obtained when *B. cenocepacia* LMG16656 was grown with *P. aeruginosa* PAO1 (Figure S3).

To test the interaction of *P. aeruginosa* and *B. cenocepacia* under attachment conditions, we challenged *P. aeruginosa* with supernatant from *B. cenocepacia*, and vice versa. Treatment with *B. cenocepacia* LMG16656 supernatant had a significant effect on attached biomass produced by *P. aeruginosa* RP73 alone (*P*<0.01) even though it did not reach the value found in the presence of living cells. Similarly, attachment by the environmental *P. aeruginosa* significantly increased when cultures were supplemented with extracellular products of environmental *B. cenocepacia* (Figure 2) as well as when *P. aeruginosa* PAO1 cultures were supplemented with extracellular products of *B. cenocepacia* LMG16656 (Figure S3).

**Figure 3:**
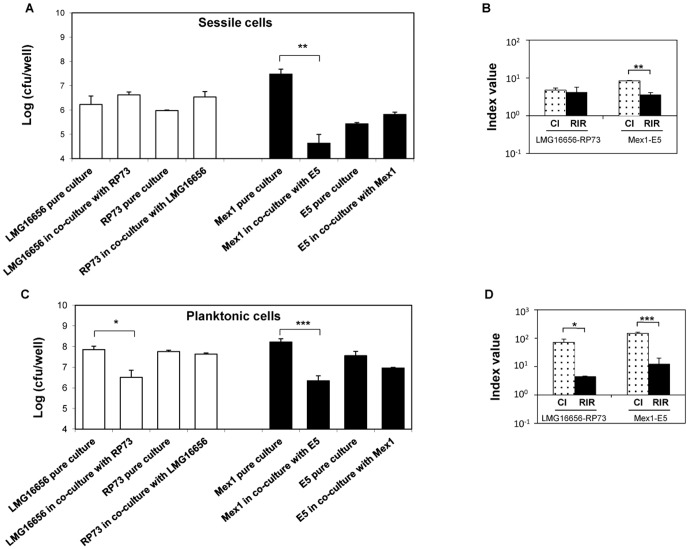
*P. aeruginosa* and *B. cenocepacia* planktonic and sessile cells in single and dual cultures. Bacteria were grown overnight in 96-well polyvinyl chloride flat-bottomed microtiter plates in NB medium at 37°C either individually cultured or co-cultured at a 1∶1 ratio. CFU counts were determined at 24 h of bacterial growth in both planktonic and sessile fraction. Key: (A, left) Sessile cells of clinical pair (*P. aeruginosa* RP73 and *B. cenocepacia* LMG16656) in single and dual cultures; (A, right) Sessile cells of environmental pair (*P. aeruginosa* E5 and *B. cenocepacia* Mex1) in single and dual cultures; (B, left) Planktonic cells of clinical pair (*P. aeruginosa* RP73 and *B. cenocepacia* LMG16656) in single and dual cultures; (B, right) Planktonic cells of environmental pair (*P. aeruginosa* E5 and *B. cenocepacia* Mex1) in single and dual cultures; (C) CI and RIR mean values of sessile growth of *P. aeruginosa* versus *B. cenocepacia* (RP73 *versus* LMG16656, E5 *versus* Mex1); (D) CI and RIR of planktonic growth of *P. aeruginosa* versus *B. cenocepacia*. Each value represents the mean of RIR and CI values from three separate assays, and the bars indicate standard deviations. * = *P*<0.05, ** = *P*<0.01, *** = *P*<0.001 in Student's t test.

To determine the amount of each bacterium attached to the microtiter plates, we performed viable counts of bacteria detached from the wells of polystyrene plates (sessile cells) after an overnight incubation (Figure 3A, B). Data showed that the clinical *P. aeruginosa* RP73 and *B. cenocepacia* LMG16656 strains were present in mixed biofilm at roughly the same concentrations as found in pure culture, respectively (*P*>0.05) (Figure 3A). Results obtained from the liquid (planktonic) fraction confirmed the previous findings from batch co-cultures experiments, where a dominant negative effect of *P. aeruginosa* RP73 on *B. cenocepacia* LMG16656 growth was found (Figure 3C,D). Very similar results were obtained when *B. cenocepacia* LMG16656 was grown with *P. aeruginosa* PAO1 (Figure S4). When we examined the paired environmental strains we found a dominant negative effect of *P. aeruginosa* E5 on both planktonic and sessile cells of *B. cenocepacia* Mex1 (*P*<0.05) (Figure 3). Overall, these data indicate that *B. cenocepacia* positively affected *P. aeruginosa* attachment to microtiter plates, and that *B. cenocepacia* secreted products may play a role in increased attachment.

### *B. cenocepacia* influences biofilm formation by *P. aeruginosa*

**Figure 4:**
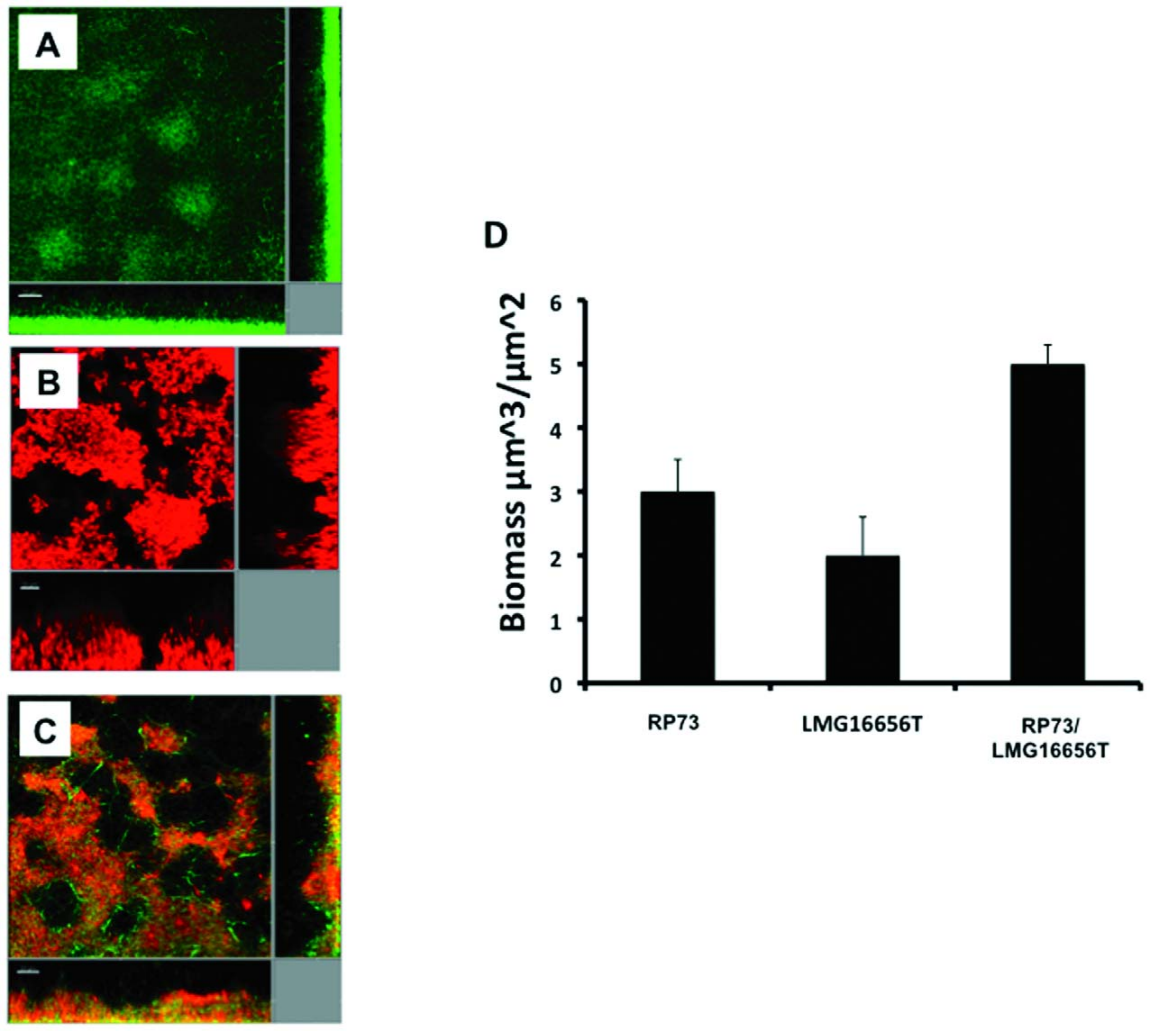
Biofilm architecture in *P. aeruginosa* is influenced by *B. cenocepacia*. Images are of 4-day-old biofilms in flow cells in FABL medium. Key: (A) *P. aeruginosa* RP73; (B) *B. cenocepacia* LMG16656;(C) mixed culture of *P. aeruginosa* RP73 and *B. cenocepacia* LMG16656; (D) Quantification of biomass as determined using COMSTAT to estimate the percentage of *P. aeruginosa* cells as a function of the total biomass. For these experiments, *P. aeruginosa* was tagged with mini-Tn*7gfp*. *B. cenocepacia* was visualized with Syto62, as described in *Materials and Methods*. Scale bars = 20 µm. Images shown are representative of 12 images from three independent experiments.

Biofilm formation was examined in clinical *P. aeruginosa* and *B. cenocepacia* strains grown in flow cells irrigated with cultures in FABL medium both singly and in combination. For these experiments, *P. aeruginosa* strains were tagged with mini-Tn*7gfp* and *B. cenocepacia* strains were visualized with Syto62. Image processing software was used to remove the Syto62 signal from the green fluorescent protein (GFP) signal of the *P. aeruginosa* cells. When grown alone, *B. cenocepacia* formed biofilms with a large microcolonies, whereas *P. aeruginosa* formed flat biofilms with little heterogeneity. When grown in co-culture, however, a significant alteration was evident in *P. aeruginosa* developed structures with a filamentous architecture within a mixed biofilm (Figure 4).

In order to quantify and compare the biofilm structures formed by the *P. aeruginosa* strains examined in the present study, we used COMSTAT software. *P. aeruginosa* biofilms showed significant structural differences in the presence of *B. cenocepacia* (Table 1). The biomass, substratum coverage, average thickness, maximum thickness and surface area of the biomass all increased for biofilms grown in the presence of *B. cenocepacia* (Table 1). Together, these findings show that co-cultivation of *B. cenocepacia* influences biofilm formation by *P. aeruginosa* leading to altered biofilm architecture and increased biomass under the conditions tested.

**Table 1:** Characteristics of *P. aeruginosa*, *B. cenocepacia* and dual culture biofilm formation as measured by COMSTAT.

Strain	Biomass	Average thickness	Roughness
	(µm^3^/µm^2^)^a^	(µm)^b^	coefficient^c^
**RP73**	3.0 (0.5)	14.1 (1.2)	0.11 (0.04)
**LMG16656^T^**	2.2 (0.4)	11.9 (2.4)	0.22 (0.08)
**RP73/LMG16656^T^**	5.3 (0.3)	26.3 (3.2)	0.62 (0.11)

a, b, c Mean (standard deviation) taken from 10 image stacks.

### Competition between *P. aeruginosa* and *B. cenocepacia* in a mouse model of chronic lung infection

To test whether the observed differences in planktonic growth and biofilm formation *in vitro* would equate to similar changes in an *in vivo* pathogenesis model, C57Bl/6NCrlBR mice were challenged with *P. aeruginosa* and *B. cenocepacia* embedded in agar beads by intratracheal inoculation. The effect of clinical (RP73- LMG16656) and environmental (E5-Mex1) pairs of strains in comparison with single infection on body weight over a 13-day period is shown in Figure S5. Mice infected with *P. aeruginosa* alone or paired with *B. cenocepacia* lost significantly more weight (and gained less weight) than mice infected with *B. cenocepacia* alone, regardless of the strains origin.

**Figure 5:**
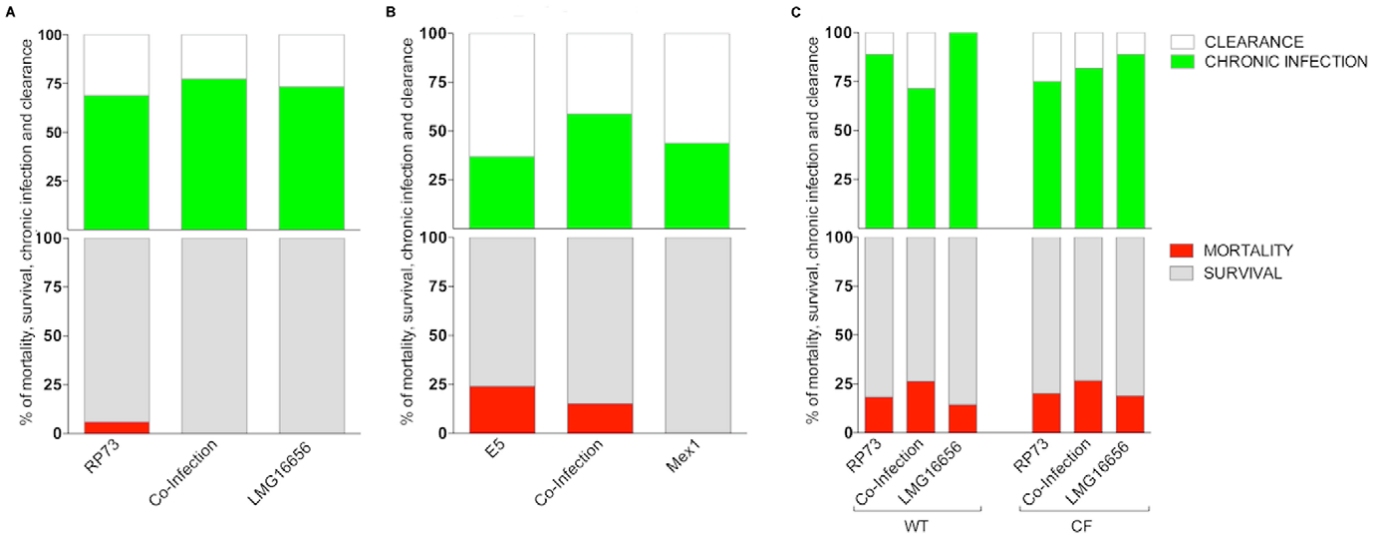
Virulence of *P. aeruginosa* and *B. cenocepacia* strains alone or in co-infection in mice. C57Bl/6 mice (A and B), *Cftr^tm1UNC^TgN(FABPCFTR)* (CF) and their congenic wt mice (C) were infected with *P. aeruginosa* and/or *B. cenocepacia* strains. Mortality induced by bacteremia (red) and survival (grey) were evaluated on challenged mice. Clearance (white) and capacity to establish chronic airways infection (green) after 13 days from challenge were determined on surviving mice infected with *P. aeruginosa* and *B. cenocepacia* strains alone or with pairs of clinical (A and C) or environmental (B) strains. The data are pooled from two to three independent experiments. Mortality and chronic infection are reported as median values. B6.129P2-*Cftr^tm1UNC^TgN(FABPCFTR) Cftr* ^+/+^ and B6.129P2-*Cftr^tm1UNC^TgN(FABPCFTR)Cftr^S489X/S489X^* mice co-infected with RP73-LMG16656 developed a higher rate of mortality when compared with C57BL/6NCrlBR mice (*P*<0.05; see Table S1).

We measured mortality induced by bacteremia *versus* mice survival and bacterial persistence *versus* clearance as readouts of virulence in the agar bead mouse model. Mortality was low and occurred within the first 3 days of infection only in the presence of *P. aeruginosa*, with no significant difference between environmental and clinical strains in single and dual-species infection (*P*>0.05) (Figure 5A, B and Table S1). When it occurred, bacteremia in co-infected mice was induced by both pathogens as indicated by similar bacterial load found in liver, kidney, spleen, and lungs in moribundus mice (data not shown).

After 13 days of challenge, chronic infection was established in C57Bl/6NCrlBR mice infected by *P. aeruginosa* and *B. cenocepacia* alone or co-infected with mixed cultures with no significant difference between the clinical and environmental pairs (*P*>0.05) (Figure 5A, B). However, only *P. aeruginosa* was recovered from the lungs of mice co-infected with *B. cenocepacia* strain (RP73 *versus* LMG16656: CFU/lung 7,8×10^4^ *versus* 0; E5 *versus* Mex1: CFU/lung 5.4×10^4^ *versus* 0) (Table S1). Overall, these results indicate that *P. aeruginosa* did not change its ability to establish chronic infection in the presence of *B. cenocepacia*. However, *B. cenocepacia* appeared to have difficulty in colonising the mouse lung during co-infection.

To test whether the microbial behavior observed in C57BL/6NCrlBR mice was also detectable in mice of other genetic backgrounds, including those that had a defective cystic fibrosis transmembrane conductance regulator (CFTR) gene, we examined clinical isolates in B6.129P2-Cftr^tm1UNC^ backcrossed into the C57Bl/6J background. Overall, it appeared that mortality was similar in both wild-type [C57BL/6NCrlBR and B6.129P2-*Cftr^tm1UNC^* TgN(FABPCFTR) *Cftr* ^+/+^] and CFTR genetic [B6.129P2-*Cftr^tm1UNC^* TgN(FABPCFTR) *Cftr^S489X/S489X^*] backgrounds when infected by RP73 and LMG16656 alone. However, RP73-LMG16656 dual-infection was significantly more virulent with regard to mortality in gut-corrected CFTR deficient mice and their congenic counterpart when compared with C57BL/6NCrlBR mice (*P*<0.05) (Figure 5C and Table S1). Chronic infection was similar in all genetic backgrounds tested. However, in gut-corrected CFTR deficient mice and their congenic counterpart *B. cenocepacia* was recovered from the lungs of co-infected mice as a rare event (one mouse out of nine was chronically infected with both *P. aeruginosa* RP73 and *B. cenocepacia* LMG16656) (Table S1). Taken together, these results confirm that chronic infection seems to be unaffected by the mouse genetic background and CFTR mutation while the host may influence the outcome of the disease in term of acute virulence.

### Host inflammatory response in mice infected with single and dual-species *P. aeruginosa* and *B. cenocepacia*

**Figure 6:**
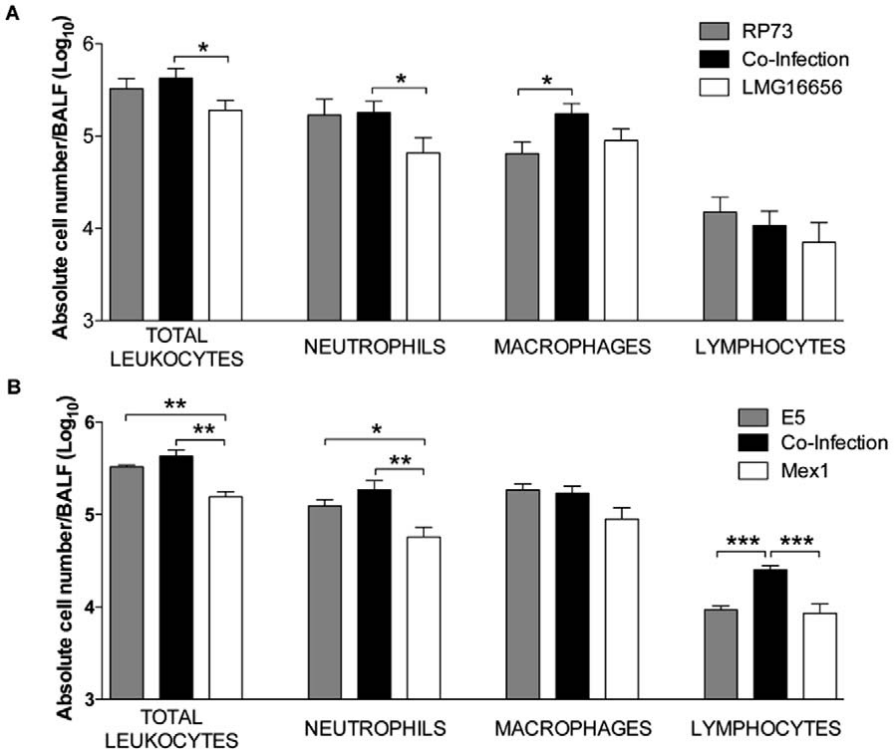
Total and differential cell counts in BAL fluid after 13 days of infection. The number of total leukocytes and in particular of neutrophils, monocytes and lymphocytes recruited in the airways were analyzed in BAL fluid (BALF) after 13 days of chronic lung infection with pairs of clinical (A) or environmental strains (B)). Values represent the mean ± SEM. The data are pooled from two or three independent experiments. Statistical significance by two tailed Student's *t*-test is indicated: * *P*<0.05, ** *P*<0.01.

The inflammatory response of mice challenged with *P. aeruginosa* and *B. cenocepacia* alone or in co-infection, in terms of total leukocytes recruitment in the airways and cytokine production, was investigated. After 13 days of infection, mice challenged with clinical (RP73- LMG16656) pairs of strains had significantly more total leukocytes in their BALF compared to mice infected with *B. cenocepacia* LMG16656 alone (*P* = 0.0332) (Figure 6A). In particular, we observed a significantly higher number of neutrophils in co-infected mice compared to mice infected with *B. cenocepacia* alone (*P* = 0.0043). Macrophages were also significantly higher in number in co-infected mice compared to mice infected with *P. aeruginosa* alone (*P* = 0.0335).

When infection was carried out with environmental (E5-Mex1) pairs of strains, mice co-infected had a higher number of total leukocytes recruited in their BALF than mice infected with *B. cenocepacia* Mex1 (*P* = 0.0018) alone (Fig. 6B), confirming and strengthening the results of clinical strains. A significantly higher number of neutrophils was observed in co-infected mice compared to mice infected with *B. cenocepacia* alone (*P* = 0.0099), while the recruitment of lymphocytes was significantly different from mice infected with *P. aeruginosa* and *B. cenocepacia* alone (*P* = 0.0001 and *P* = 0.0006, respectively). No significant differences were observed in the recruitment of macrophages.

Finally, we measured the concentration of cytokines and chemokines in the murine lungs. When we evaluated the clinical pair RP73-LMG16656, the expression of pro-inflammatory cytokine IL-1β and chemokines CCL2/JE and CXCL1/KC (homologues of human IL-8) in co-infected mice were significantly higher than in those infected with single strains alone [IL-1β: *P* = 0.0477 (mixed *versus* RP73) and *P* = 0.0110 (mixed *versus* LMG16656); CCL2/JE: *P* = 0.0027 (mixed *versus* RP73) and *P* = 0.0024 (mixed *versus* LMG16656); CXCL1/KC: *P* = 0.0087 (mixed *versus* RP73) and *P* = 0.0152 (mixed *versus* LMG16656)], while no difference in the level of CXCL2/MIP-2 was found (Table 2). In the case of environmental strains E5-Mex1, a significantly higher level of the pro-inflammatory cytokine IL-1β was seen in co-infected mice compared to mice infected with single species alone [*P* = 0.0586 (mixed *versus* E5) and *P* = 0.0134 (mixed *versus* Mex1)] while no differences in the level of CXCL1/KC and CXCL2/MIP-2 between co-infection and single infections were observed (Table 2). In addition, the chemokine CCL2/JE was significantly higher in co-infected mice than in those infected with *B. cenocepacia* alone (*P* = 0.0472). Overall, these data show that *P. aeruginosa* and *B. cenocepacia* co-infection could affect the host response by increasing airway inflammation in respect to single infection.

**Table 2:** Cytokines and chemokines in lungs homogenates of C57BL/6NCrlBR mice after chronic infection.

Bacterial strains	IL-1β^a^	CCL2/JE^a^	CXCL1/KC^a^	CXCL2/MIP-2^a^
**RP73** b	402±94^#^	28±2^##^	190±16^##^	106±24
**Co-infection**	799±138	51±5	413±54	130±41
**LMG16656** c	293±82^Δ^	31±2^ΔΔ^	225±37^Δ^	76±34
**E5**	395±102	38±3	242±67	302±64
**Co-infection**	696±101	38±5	202±24	150±35
**Mex1**	291±96^Δ^	24±2^Δ^	252±60	147±28

a Cytokines and chemokines are expressed as pg/ml (mean±SEM).

b Significant difference of *P. aeruginosa* RP73 single infection *vs* co-infected mice (^#^ *P*<0.05;^# #^ *P*<0.01 Two-tailed student's t test).

c Significant difference of *B. cenocepacia* LMG16656 single infection *vs* co-infected (^Δ^ *P*<0.05; ^ΔΔ^ *P*<0.01 Two-tailed student's t test).

## Discussion

CF lung infections often involve more than one microbial species. The complex microbial communities of the CF respiratory tract provide an environment in which a number of bacterial species can interact and face continuous adaptative challenges. The significance of microbe-microbe interactions and the interplay of these communities with the host remain poorly understood. The goal of this study was to examine the interactions between *P. aeruginosa* and *B. cenocepacia* in co-culture, during biofilm development and mouse colonization, with the objective of better understanding polymicrobial infection and its possible role in pathogenesis. Furthermore, since both *P. aeruginosa* and *B. cenocepacia* are capable of adapting and controlling their development to suit the various environments they find themselves in [17], [18] and are constantly evolving under selective pressures [19], [20], we examined a selection of clinical and environmental isolates in this study. The presented data show that the co-infection of *P. aeruginosa* and *B. cenocepacia*, particularly the clinical pair, regardless of the isolates origin, leads to increased biofilm formation and increased host inflammatory response in CF and non-CF mouse models of chronic infection. Overall, these results suggest that alteration of bacterial behaviour due to interspecies interactions may be important for disease progression.

Studies into microbial interactions during growth in complex media has already revealed that there are diverse mechanisms by which species can co-exist with other microorganisms competing for the same pool of resources [21]. It has been demonstrated that the consumption of limited nutrients can shape the course of interaction between bacteria in co-culture [6], [22]. Here we have shown that the bacterial growth of *P. aeruginosa* in rich medium was not affected by the presence of *B. cenocepacia*, whilst a dominant negative effect of *P. aeruginosa* over *B. cenocepacia* growth was found at the end of logarithmic phase and during the stationary-phase of bacterial growth. This may involve simple resource competition or direct antagonistic effects [22]. Examination of spent culture supernatants, suggest that secreted compounds from *P. aeruginosa* could account for the disadvantage of *B. cenocepacia* in planktonic cultures when grown in co-culture with *P. aeruginosa*. This is consistent with previous work by Al-Bakri and colleagues [23] who suggested that various *P. aeruginosa* secondary metabolites were functioning as inhibitory factors of *B. cenocepacia* growth.

An important feature of *P. aeruginosa* and *B. cenocepacia* infection is their ability of form biofilms, which is one of the contributing factors to reduced antibiotic efficacy and poor patient prognosis [24], [25]. Many properties of mono-species biofilms and their role in CF disease have been extensively studied [26]. However, the polymicrobial interactions within mixed biofilms have been examined in a small selection of studies associated with CF infection [27]–[29]. Here we demonstrated the ability of both clinical and environmental isolates of *B. cenocepacia* to develop mixed biofilms with various strains of *P. aeruginosa*. Previous work has revealed that *B. cepacia* and *P. aeruginosa* strains, when simultaneously introduced into an un-colonized surface, produce a stable mixed biofilm [23]. Our work builds on this finding by showing that the development of synergistic biofilms between various clinical strains of *B. cenocepacia* and *P. aeruginosa* leads to the generation of greater biomass than their mono-culture counterparts. Our results confirm that the biomass and the fitness of dual-species biofilm are not necessarily the sum of the characteristics of each single species. This is consistent with the emerging theme that some bacterial communities associated with chronic infection are gaining a fitness advantage from residing in multispecies biofilms [30].

As well as with the development of biofilms, interactions between microorganisms have been reported to influence virulence factor production and pathogenicity [6], [31]. To investigate this in the context of the chronically infected CF lung we used the agar bead mouse model to characterize interactions between *B. cenocepacia* and *P. aeruginosa*. In this case to mimic the bronchopulmonary infection typical of CF patients, long-term chronic infection was established in mice [32], [33]. Our data have shown, that both clinical and environmental *P. aeruginosa* and *B. cenocepacia* strains, in single and dual-infection, induce mortality and chronic infection two weeks after bacterial challenge. No difference in the rate of induction of bacteremia in mice infected with single or both *P. aeruginosa* and/or *B. cenocepacia* was observed. However, our results did indicate that inflammation markers were increased by co-infection suggesting a significant contribution to lung damage by dual species than single one. This may impact the clinical disease in CF patients. Interestingly, in our experiments total bacterial numbers, recovered from the murine airways, did not differ between single and dual-infection, indicating that the greater inflammatory response due to dual-infection in murine airways could be influenced by virulence factor production in synergistic biofilms rather than by the amount of bacteria. This scenario is similar to that of pathogens belonging to other microbial species in other clinical disease [34], [35].

Because virulence of a given pathogen is dependent on a specific host, we investigated interactions between *B. cenocepacia* and *P. aeruginosa* in other genetic backgrounds including CFTR deficient mice. The rate of chronic infection in single or co-infected mice seems to be unaffected by the mouse genetic background and CFTR mutation. This is consistent with our recent studies showing that long term chronic infection in murine lungs is established regardless of the CF genetic background [36]. However, the B6.129P2-Cftr^tm1UNC^ backcrossed into the C57Bl/6J background develop a higher rate of mortality in co-infection (RP73-LMG16656) when compared with C57BL/6NCrlBR mice suggesting that the host may influence the outcome of the disease in term of acute virulence.

It is important to note that co-infections with equal ratios of *P. aeruginosa* and *B. cenocepacia* most likely never occur in a CF lung where long standing *P. aeruginosa* infection with high levels are present when *B. cenocepacia* arrives. Further experiments are required to tease such scenarios out, for example, where *B. cenocepacia* would be added to already established chronic *P. aeruginosa* infection in order to mirror what occurs during progression of CF infection. In addition, given the diversity of bacterial strains associated with colonisation of the CF airway, further combinations of strains will need to be tested, particularly strains that have been residing in the airway for different lengths of time.

In conclusion, understanding the microbial community of the CF airway is of considerable importance as interactions between community members can potentially affect biofilm formation and virulence of pathogens. It is clear from our experiments that the complex interactions between bacteria in the host play important roles in this complex disease. Furthermore, these observations point towards the growing opinion that greater characterization and management of CF disease as a polymicrobial infection may unveil alternative treatment strategies.

## Materials and Methods

### Ethics Statement

Animal studies were carried out in strict accordance with the Italian Ministry of Health guidelines for the use and care of experimental animals. This study was approved by the San Raffaele Scientific Institute (Milan, Italy) Institutional Animal Care and Use Committee (IACUC, Number 369). All efforts were made to minimize the number of animals used and their suffering.

### Bacterial strains

We investigated *B. cenocepacia* and *P. aeruginosa* strains before they managed to adapt to the CF niche, thus by examining a pair of ‘pristine’ environmental strains, and after they have adapted to the CF niche, thus by examining a pair of typical CF isolates. Two *P. aeruginosa* and two *B. cenocepacia* strains of clinical and environmental origin were used in this study: the non-mucoid clinical *P. aeruginosa* RP73 strain, isolated at the late stage of chronic infection from a CF patient [37]; the environmental *P. aeruginosa* E5 strain, isolated from red pepper [32]; the *B. cenocepacia* Mex1 strain, belonging to *recA* lineage IIIA, isolated from the maize rhizosphere in Mexico [38], [39]; and the epidemic and fully sequenced *B. cenocepacia* LMG16656^T^ strain (ET12 reference strain), *recA* lineage IIIA, isolated from a CF patient [40]. Strains were selected according to their origin and their similar degree of pathogenicity *in vivo* [32], [37], [38]. The *P. aeruginosa* RP73 and E5 strains were kindly provided by Prof. Burkhard Tummler (Klinische Forschergruppe, Medizinische Hochschule, Germany) and Gerd Doring (Institut für Medizinische Mikrobiologie und Hygiene, Germany). *B. cenocepacia* strain Mex 1 was kindly provided by Jesus Caballero-Mellado (Universidad Nacional Autonoma de Mexico, Cuernavaca, Mexico). *B. cenocepacia* LMG16656 (strain J2315) was obtained from BCCM/LMG Culture Collection, Laboratorium voor Microbiologie, Gent, Belgium. All strains were stored at −80°C in 30% (v/v) glycerol. Prior to assays, bacteria were streaked from frozen stock preparations onto Nutrient Agar (NA, Difco) plates and incubated at 30°C for 24–48 h.

### Planktonic mono-culture and co-culture growth curves

All growth cultures were performed in Nutrient Broth (NB, Difco™) at 37°C and performed in triplicate. Co-cultures (50 ml) were inoculated by pure cultures of *P. aeruginosa* or *B. cenocepacia* grown to mid-exponential phase (OD_600_∼0.5–0.6), diluted to OD_600_ = 0.025, and combined in a 1∶1 ratio. Pure cultures of each organism was used for comparative purposes. At different growth stages (0, 2, 4, 6, 8 and 24 h), samples were serially diluted in sterile phosphate-buffered saline (PBS), plated on Tryptic Soy Agar (TSA) (Difco™) and incubated for 48 h at 37°C. On TSA, the two species were easily distinguished from each other by the appearance of their colonies and the difference in growth; i.e., *P. aeruginosa* colonies appeared after 24 h of incubation while *B. cenocepacia* colonies after 48–72 h. *B. cenocepacia* cells were also enumerated by plating serial dilutions of the planktonic co-cultures on *Burkholderia cepacia* selective agar (BCSA). The generation time of bacteria was calculated using the following equations: *K* = [Log (CFU/ml) _T5_−Log (CFU/ml) _T2_]/[0,301×h (T5-T2)], t = 1/k, where *K* is the number of generations occurring during the exponential phase of growth, T is the time, and *t* is the generation time. Competitive index (CI) and Relative Increase Ratio (RIR) were calculated, as described by Macho and colleagues [41], by using colony forming unit (CFU) counts resulting from the growth of each strain in co-cultures and pure-cultures, respectively. The CI (CI = *P. aeruginosa*-to-*B. cenocepacia* ratio within the output sample, divided by the corresponding ratio in the inoculum, using growth results from co-cultures) was calculated at 4, 8, and 24 h. Mono-cultures for each organism were performed to calculate the RIR (RIR = *P. aeruginosa*-to-*B. cenocepacia* ratio within the output sample, divided by the corresponding ratio in the inoculum, using growth results from individual inoculations of each strain). Each value represents the mean of RIR and CI values from three separate assays, and the bars indicate standard deviations. * = P<0.05, ** = P<0.01 in the Student's t test.

### Preparation of supernatants of *P. aeruginosa* and *B. cenocepacia* strains

Overnight cultures (∼16 h) of *P. aeruginosa* and *B. cenocepacia* in 50 ml of NB were centrifuged at 8,000×g at 4°C and the crude supernatant was filtered through a 0.2-µm-pore-size filter (Millipore, Bedford, MA, USA). To ensure that no cells were present in the filtrates, 100 µl of the crude supernatant was spread onto NA agar plates, and the remaining supernatant was lyophilized aseptically on a Edwards Modulyo freeze dryrer (Edwards High Vacuum Ltd.). The dried supernatants from *B. cenocepacia* or *P. aeruginosa* cultures were resuspended in sterile distilled water to a 50-fold concentration and filter sterilized. As a control, sterile NB media was filtered, lyophilized and resuspended to a concentration of 50× in the same manner as the bacterial samples.

### Planktonic growth of *P. aeruginosa* in the supernatant of *B. cenocepacia* and vice versa

An overnight culture of *P. aeruginosa* or *B. cenocepacia* was diluted to OD_600_ = 0.025 in 50 ml fresh NB. Immediately prior to the inoculation, sterile concentrated bacterial supernatant (50× stock) obtained from the counterpart organism was added to a final concentration of 1×, and the flasks were incubated at 37°C with shaking. As controls, pure cultures were grown in NB medium alone and in NB medium supplemented with concentrated NB medium to a final concentration of 1×. Aliquots were removed aseptically at defined time intervals (2, 4, 6, 8 and 24 h) and the OD_600_ was measured.

### Quantification of attachment in microtiter plates

#### Crystal violet assay

Biofilm production assay was performed as described previously [42], with minor modifications. The method used was based on staining biofilms with crystal violet (CV). Briefly, *B. cenocepacia* and *P. aeruginosa* strains were inoculated individually or at equal ratio (1∶1) from pure cultures grown in NB to mid-exponential (OD_600_∼0.5) phase into at least 6 wells of flat-bottomed 96-well polyvinylchloride microtiter plates (Greiner Bio-one, Frickenhausen, Germany). The final volume added to each well was 200 µl. Pure cultures of each organism were performed for comparative purposes. Plates were then sealed with Parafilm and incubated with shaking (100 rpm) at 37°C for 24 h. Then, the planktonic cell fractions of pure cultures and co-cultures were transferred to new microtiter plates while the attached cells were rinsed three times with 200 µl of phosphate buffered saline (PBS) to remove non-adherent and weakly adherent bacteria. Then, plates were air dried for 30 min before addition of 200 µl of 1% (w/v) crystal violet (CV). After 20 min of staining at room temperature, the excess CV was removed by washing the wells three times with 200 µl of PBS. The bound dye was dissolved using 200 µl of 95% (v/v) ethanol and absorbance at 595 nm was determined with a Victor^3^ Multilabel Counter (Perkin Elmer). Experiments were performed in triplicate and repeated in three independent experiments. The data was then averaged and the standard deviation was calculated. To compensate for background absorbance, OD readings from sterile medium, dye and ethanol were averaged and subtracted from all test values.

#### Planktonic and sessile cells

To correlate biofilm formation with the growth of planktonic *P. aeruginosa* and *B. cenocepacia* cells in each well, the planktonic cell fractions, which were transferred to new microtiter plates following 24 h of growth, were quantified by plating serial dilutions on TSA and BCSA agar plates. To enumerate the sessile (adhered) cells of *B. cenocepacia* and *P. aeruginosa*, the wells were rinsed three times with 200 µl of PBS to remove non-adherent and weakly adherent bacteria. Then, the biofilm was removed by scraping the surface of each well with 1 ml PBS and the recovered cells were suspended by vortexing for 30 sec. The number of sessile cells was determined by plating appropriate dilutions of biofilm samples on BCSA and TSA media. To ensure the complete detachment of the bacteria, CV (1%) assay was performed on each of the wells scraped, and absorbance determined at 595 nm.

#### Effect of supernatant of P. aeruginosa on biofilm formation of B. cenocepacia and vice versa

*B. cenocepacia* or *P. aeruginosa* strains were inoculated from pure cultures grown in NB to mid-exponential (OD_600_∼0.5) phase into at least 6 wells of flat-bottomed 96-well polyvinylchloride microtiter plates. Sterile concentrated bacterial supernatants were added into the wells to a final concentration of 1× from a 50× stock. The final volume added in each well was 200 µl. Cultures with no added supernatants were used as controls. Biofilm formation was examined by CV (1%) assay as described previously. Fresh growth medium plus 1× supernatant was added to the wells in order to obtain a background value, which was subtracted from values obtained from the wells containing cells. Plates were then sealed with Parafilm and incubated with shaking (100 rpm) at 37°C for 24 h.

### Cultivation of biofilms

Biofilms were grown in three-channel flow cells with individual channel dimensions of 1×4×40 mm. The flow system was assembled and prepared as described previously [43], [44], with the modification of washing the system after sterilization with sterile milliQ water overnight. The substratum consisted of a microscope glass coverslip. Each channel was supplied with a continuous flow of FABL medium containing the relevant carbon source. For propagation of mixed-species biofilm populations, flow cells were inoculated with a mixture of overnight cultures of *P. aeruginosa* and *B. cenocepacia* diluted in a 0.9% NaCl solution.

For monospecies biofilms, overnight cultures of the *P. aeruginosa* and *B. cenocepacia* were used for inoculation. With arrested medium flow, the flow cells were turned upside down, and 250 µl of the diluted mixture was injected into each flow channel, using a small syringe. After 1 h, the flow cells were turned upside down, and the flow was resumed at a constant flow rate of 3.3 ml/h, using a Watson Marlow 205S peristaltic pump (Watson Marlow Inc., Wilmington, MA). After inoculation, each flow chamber contained 2×10^6^ CFU of *Pseudomonas* and 2.5×10^5^ CFU of *B. cenocepacia* for mixed-species biofilms and 2.5×10^5^ CFU of *Pseudomonas* for monospecies biofilms. The mean flow velocity in the flow cells was 0.2 mm/s. Biofilms were grown at 30°C. When possible, *B. cenocepacia* was visualized prior to image acquisition by staining the biofilm with a 0.1% solution of Syto62 in FABL medium containing 500 µM benzyl alcohol. The staining was allowed to progress for 15 min without arresting the flow to avoid biofilm detachment of the *Pseudomonas* strain. Using this relatively short staining time, *Pseudomonas* cells were stained at a relatively low level compared to *B. cenocepacia* cells.

### Microscopy and image analysis of biofilms

All microscopic observations and image acquisitions were performed on a Zeiss LSM510 confocal laser scanning microscope (CSLM; Carl Zeiss, Jena, Germany) equipped with an argon-krypton laser and with detectors and filter sets for monitoring green fluorescent protein (GFP) and Syto62 and for the recording of reflection (light) images. Images were obtained using a 63×/1.4 Plan-APOChromat differential interference contrast objective or a 40×/1.3 Plan-Neofluor oil objective. Multichannel simulated fluorescence projection (SFP) images, vertical *xz* sections through the biofilms, and simulated three-dimensional (3D) images were generated using the IMARIS software package (Bitplane). This software was used to remove the Syto62 signal from the GFP-fluorescent PAO1 cells. Images were further processed for display using Photoshop software (Adobe, Mountain View, CA). Biofilm images of the mixed-species consortia were obtained to quantify biomass as described previously [43], [44], using COMSTAT software. Twelve images from three independent biofilms were analyzed for each time point.

### Mouse strains

C57Bl/6NCrlBR male mice, 6–8 weeks (Charles River), congenic gut-corrected CFTR deficient mice (B6.129P2-Cftr^tm1UNC^TgN(FABPCFTR), Case Western Reserve University) and homozygotes male and female, 5–16 weeks [45] were used. C57Bl/6NCrlBR mice were fed with standard rodent autoclaved chow (4RF21-GLP, Mucedola) while Cftr^S489X/S489X^ and Cftr^+/+^ mice were fed with high-fat rodent diet (Teklad 2019, Harland) and autoclaved tap water. All mouse strains were maintained in specific pathogen-free conditions.

### Preparation of agar beads

The agar-bead models of *P. aeruginosa* and *B. cenocepacia* chronic lung infection was used [32], [38], [46]. Co-infection was established by embedding *P. aeruginosa* and *B. cenocepacia* strains in agar beads at 1∶10 ratio, respectively. Briefly, *P. aeruginosa* and *B. cenocepacia* strains were grown separately overnight at 37°C to the stationary phase, in Tryptic Soy Broth (TSB) (Difco™) or NB, respectively. Then, bacteria were harvested by centrifugation and re-suspended in 1 ml of PBS (pH 7.4). A starting amount of 5×10^9^ and 5×10^10^ CFU ml^−1^ for *P. aeruginosa* and *B. cenocepacia,* respectively, was used for inclusion in the agar beads according to the previously described method. The cells were added to 9 ml of NA pre-warmed to 50°C. This mixture was pipetted forcefully into 150 ml of heavy mineral oil (Sigma Aldrich) at 50°C and stirred rapidly with a magnetic stirring bar for 6 min at room temperature, followed by cooling at 4°C with continuous slow stirring for 20 min. The oil-agar mixture was centrifuged at 4,000 rpm for 20 min to sediment the beads and washed six times in PBS. The inoculum was prepared by diluting the bead suspension with PBS to 2–4×10^7^ CFU ml^−1^ of *P. aeruginosa* and 2–4×10^8^ CFU ml^−1^ of *B. cenocepacia*, in both single and dual species infections. The number of *P. aeruginosa* and *B. cenocepacia* CFU embedded in the beads alone or in combination was determined by plating serial dilutions of the homogenized bacteria-bead suspension on TSA and BCSA plates, respectively.

### Mouse models of chronic lung single infection and co-infection

Mice were anesthetized by an intraperitoneal injection of a solution of 2.5% Avertin (2,2,2-tribromethanol, 97%; Sigma Aldrich) in 0.9% NaCl and administered in a volume of 0.015 mlg^−1^ body weight. Mice were then placed in dorsal recumbency and the trachea was directly visualized by a ventral midline incision, exposed and intubated with a sterile, flexible 22-g cannula (Becton, Dickinson, Italy) attached to a 1 ml syringe. Co-infection was established with a 100 µl inoculum of an agar bead suspension containing *P. aeruginosa* (1–2×10^6^ CFU) and *B. cenocepacia* (1–2×10^7^ CFU) at a multiplicity of infection (MOI) equal to 1∶10 (*P. aeruginosa*/*B. cenocepacia*). Mice were also infected with 1–2×10^6^ CFU ml^−1^ of *P. aeruginosa* or 1–2×10^7^ CFU ml^−1^ of *B. cenocepacia* embedded in agar beads for comparative purposes. Mice were observed daily for clinical signs, such as coat quality, posture, ambulation, hydration status and body weight. Acute infection was assessed as bacteremia in moribund mice. Lungs, spleens, kidneys and livers were excised, homogenized and plated onto TSA and BCSA plates, for *P. aeruginosa* and *B. cenocepacia*, respectively. Recovery of >1,000 CFU of bacteria from cultures of multiple organs was indicative of bacteremia. Chronic infection was evaluated in surviving mice 13 days after challenge. Mice were sacrificed by CO_2_ administration and murine lungs were removed aseptically, homogenized in PBS and plated as above reported. Recovery of >1,000 CFU from lung cultures was indicative of chronic infection.

### BALF collection and analysis

The bronchoalveolar lavage fluid (BALF) was extracted with a 22-gauge venous catheter by washing the lungs three times with 1 ml of RPMI-1640 (Euroclone) with protease inhibitors (Complete tablets, Roche Diagnostic and PMSF, Sigma). Total cells present in the BALF were counted, and a differential cell count was performed on cytospins stained with Diff Quick (Dade, Biomap, Italy). BALF was serially diluted 1∶10 in PBS and plated on TSA and BCSA plates for *P. aeruginosa* and *B. cenocepacia* CFU counts, respectively.

### Lung homogenization and cytokine analysis

Lungs were removed and homogenized in 1 ml PBS with Ca^2+^/Mg^2+^ containing protease inhibitors. Samples were serially diluted 1∶10 in PBS and plated on the above agar media for CFU counts. Lung homogenates were then centrifuged at 14,000 rpm for 30 minutes at 4°C, and the supernatants were stored at −20°C for cytokine analysis. Murine IL-1β, CCL2/JE, CXCL1/KC and CXCL2/MIP-2 were measured in the supernatants of lung homogenates by ELISA (R&D DuoSet ELISA Development System, USA), according to manufacturer's instructions.

### Statistical analysis

*In vitro* data were analyzed using Two-tailed Student's *t*-test and ANOVA, considering *P*<0.05 as the limit of statistical significance. All data were expressed as mean ± standard deviation (SD). *In vivo* data were represented as the means ± standard error medium (SEM), unless stated otherwise. Statistical testing was performed by chi-square test or Fisher's exact test (two-tailed) for categorical variables. Two-tailed Student's *t*-test was used for comparison of continuous variables. Differences were considered statistically significant at *P* values<0.05.

## Supporting Information

Figure S1 Single and dual species batch growth curves of laboratory *P. aeruginosa* PAO1 and clinical *B. cenocepacia* LMG16656 strains and the competitive index (CI) and relative increase ratio (RIR) values. (A) The two species were individually cultured or co-cultured at a 1∶1 ratio and grown for 24 h in NB medium at 37°C with vigorous aeration. Colony-forming unit counts (CFU) was determined at 0, 2, 4, 6, 8 and 24 h of bacterial growth. The results are the mean of Log (cfu/mL) values of three separate assays. Key: (A) Growth of laboratory *P. aeruginosa* PAO1 and *B. cenocepacia* LMG16656 strains in single and dual cultures; (B) CI and RIR generated from single and dual cultures of laboratory *P. aeruginosa* PAO1 and *B. cenocepacia* LMG16656 strains. CI and RIR were calculated as described in *Materials and Methods*. Each value represents the mean of RIR and CI values from three separate assays, and the bars indicate standard deviations. * = *P*<0.05, ** = *P*<0.01 in the Student's t test.
(TIF)

Figure S2 Effect of the supernatants on planktonic bacterial growth. (A) Effect of the supernatant of *P. aeruginosa* cultures on growth of planktonic cultures of *B. cenocepacia*. (B) Effect of the supernatant of *B. cenocepacia* cultures on growth planktonic cultures of *P. aeruginosa*. The two species were grown at 37°C with vigorous aeration in NB medium supplemented with sterile concentrated supernatant of the second organism at a final concentration of 1×. As controls, pure cultures were grown in NB medium alone and in NB medium supplemented with concentrated NB medium to a final concentration of 1×. OD_600_ was measured at 0, 2, 4, 6 and 8 h of bacterial growth. The means ± standard deviations for at least three separate assays are illustrated. * = P<0.05, ** = P<0.01, *** = *P*<0.001 in the Student's t test with respect to the pure cultures grown in NB medium; s = supernatant; NBc = concentrated Nutrient Broth medium.
(TIF)

Figure S3 Biofilm formation by laboratory *P. aeruginosa* PAO1 and clinical *B. cenocepacia* LMG16656 strains in single and dual cultures. Bacteria were grown overnight in 96-well polyvinyl chloride flat-bottomed microtiter plates in NB medium at 37°C either individually cultured or cocultured at a 1∶1 ratio or when individually cultured supplemented with sterile concentrated supernatant of the second organism at a final concentration of 1×. Biofilm biomass was quantified by staining with crystal violet and absorbance measurements at OD _595_. The values are means of three separate assays, and the bars indicate standard deviation. * = *P*<0.05, ** = *P*<0.01 in Student's t test. s = supernatant.
(TIF)

Figure S4 Laboratory *P. aeruginosa* PAO1 and clinical *B. cenocepacia* LMG16656 planktonic and sessile cells in single and dual cultures and the competitive index (CI) and relative increase ratio (RIR) values. Bacteria were grown overnight in 96-well polyvinyl chloride flat-bottomed microtiter plates in NB medium at 37°C either individually cultured or cocultured at a 1∶1 ratio. CFU counts were determined at 24 h of bacterial growth in both planktonic and sessile fraction. Key: (A) Planktonic (left) and sessile (right) cells of laboratory *P. aeruginosa* PAO1 and clinical *B. cenocepacia* LMG16656 in single and dual cultures; (B) CI and RIR mean values of planktonic growth of laboratory *P. aeruginosa* PAO1 versus clinical *B. cenocepacia* LMG16656. Each value represents the mean of RIR and CI values from three separate assays, and the bars indicate standard deviations. * = *P*<0.05, ** = *P*<0.01 in Student's t test.
(TIF)

Figure S5 Weight change after infection with *P. aeruginosa* and *B. cenocepacia* alone or in co-infection. C57Bl/6NCrlBR mice were infected with *P. aeruginosa*, *B. cenocepacia* strains alone or in combination and monitored for weight change. Values are the mean daily weight gain over 13-day infection. Key: (A) Co-infection with clinical strains: mice co-infected with both pathogens lost significantly more weight than mice infected with *B. cenocepacia* LMG16656 alone from days 1 to 6; (B) Co-infection with environmental strains: mice infected with *P. aeruginosa* E5 alone or in coinfection lost significantly more weight than mice infected with *B. cenocepacia* Mex1 alone.
(TIF)

Table S1 Colonization of murine lung with clinical and environmental *P. aeruginosa* and *B. cenocepacia* strains.
(DOCX)

## References

[1] Sibley CD, Rabin H, Surette MG (2006) Cystic fibrosis: a polymicrobial infectious disease. Future Microbiol 1: 53–61. 17661685 10.2217/17460913.1.1.53

[2] Rajan S, Saiman L (2002) Pulmonary infections in patients with cystic fibrosis. Semin Respir Infect 17: 47–56. 11891518 10.1053/srin.2002.31690

[3] Lipuma JJ (2010) The changing microbial epidemiology in cystic fibrosis. Clin Microbiol Rev 23: 299–323. 20375354 10.1128/CMR.00068-09 PMC2863368

[4] Gilligan PH (1991) Microbiology of airway disease in patients with cystic fibrosis. Clin Microbiol Rev 4: 35–51. 1900735 10.1128/cmr.4.1.35 PMC358177

[5] Govan JR, Deretic V (1996) Microbial pathogenesis in cystic fibrosis: mucoid Pseudomonas aeruginosa and Burkholderia cepacia. Microbiol Rev 60: 539–574. 8840786 10.1128/mr.60.3.539-574.1996 PMC239456

[6] Sibley CD, Duan K, Fischer C, Parkins MD, Storey DG, et al (2008) Discerning the Complexity of Community Interactions Using a Drosophila Model of Polymicrobial Infections. PLoS Pathog 4 10: e1000184 doi:10.1371/journal.ppat.1000184 18949036 10.1371/journal.ppat.1000184 PMC2566602

[7] Rogers GB, Carroll MP, Bruce KD (2009) Studying bacterial infections through culture-independent approaches. J Med Microbiol 58: 1401–1418. 19556372 10.1099/jmm.0.013334-0

[8] Stressmann FA, Rogers GB, Klem ER, Lilley AK, Donaldson SH, et al (2011) Analysis of the bacterial communities present in lungs of patients with cystic fibrosis from American and British centers. J Clin Microbiol 49: 281–291. 21068277 10.1128/JCM.01650-10 PMC3020463

[9] Rogers GB, Hoffman LR, Whiteley M, Daniels TW, Carroll MP, et al (2010) Revealing the dynamics of polymicrobial infections: implications for antibiotic therapy. Trends Microbiol 18: 357–364. 20554204 10.1016/j.tim.2010.04.005 PMC3034215

[10] Eberl L, Tummler B (2004) Pseudomonas aeruginosa and Burkholderia cepacia in cystic fibrosis: genome evolution, interactions and adaptation. Int J Med Microbiol 294: 123–131. 15493822 10.1016/j.ijmm.2004.06.022

[11] Whiteford ML, Wilkinson JD, McColl JH, Conlon FM, Michie JR, et al (1995) Outcome of Burkholderia (Pseudomonas) cepacia colonisation in children with cystic fibrosis following a hospital outbreak. Thorax 50: 1194–1198. 8553277 10.1136/thx.50.11.1194 PMC475093

[12] McCloskey M, McCaughan J, Redmond AO, Elborn JS (2001) Clinical outcome after acquisition of Burkholderia cepacia in patients with cystic fibrosis. Ir J Med Sci 170: 28–31. 11440408 10.1007/BF03167716

[13] Jones AM, Dodd ME, Govan JR, Barcus V, Doherty CJ, et al (2004) Burkholderia cenocepacia and Burkholderia multivorans: influence on survival in cystic fibrosis. Thorax 59: 948–951. 15516469 10.1136/thx.2003.017210 PMC1746874

[14] Sibley CD, Surette MG (2011) The polymicrobial nature of airway infections in cystic fibrosis: Cangene Gold Medal Lecture. Can J Microbiol 57: 69–77. 21326348 10.1139/w10-105

[15] Zemanick ET, Sagel SD, Harris JK (2011) The airway microbiome in cystic fibrosis and implications for treatment. Curr Opin Pediatr 23: 319–324. 21494150 10.1097/MOP.0b013e32834604f2

[16] Zhao J, Schloss PD, Kalikin LM, Carmody LA, Foster BK, et al (2012) Decade-long bacterial community dynamics in cystic fibrosis airways. Proc Natl Acad Sci U S A 109: 5809–5814. 22451929 10.1073/pnas.1120577109 PMC3326496

[17] Coggan KA, Wolfgang MC (2012) Global regulatory pathways and cross-talk control Pseudomonas aeruginosa environmental lifestyle and virulence phenotype. Curr Issues Mol Biol 14: 47–70. 22354680 PMC12747716

[18] Vial L, Chapalain A, Groleau MC, De' ziel E (2011) The various lifestyles of the Burkholderia cepacia complex species: a tribute to adaptation. Environ Microbiol 13: 1–12. 20880095 10.1111/j.1462-2920.2010.02343.x

[19] Mira NP, Madeira A, Moreira AS, Coutinho CP, Sá-Correia I (2011) Genomic expression analysis reveals strategies of Burkholderia cenocepacia to adapt to cystic fibrosis patients' airways and antimicrobial therapy. PLoS ONE 6 12:e28831 doi:10.1371/journal.pone.0028831. 22216120 10.1371/journal.pone.0028831 PMC3244429

[20] Yang L, Jelsbak L, Marvig RL, Damkiaer S, Workman CT, et al (2011) Evolutionary dynamics of bacteria in a human host environment. Proc Natl Acad Sci USA 108: 7481–7486. 21518885 10.1073/pnas.1018249108 PMC3088582

[21] Hibbing ME, Fuqua C, Parsek MR, Peterson SB (2010) Bacterial competition: surviving and thriving in the microbial jungle. Nat Rev Microbiol 8: 15–25. 19946288 10.1038/nrmicro2259 PMC2879262

[22] Harrison F (2007) Microbial ecology of the cystic fibrosis lung. Microbiology 153: 917–923. 17379702 10.1099/mic.0.2006/004077-0

[23] Al-Bakri AG, Gilbert P, Allison DG (2004) Immigration and emigration of Burkholderia cepacia and Pseudomonas aeruginosa between and within mixed biofilm communities. J Appl Microbiol 96: 455–463. 14962125 10.1111/j.1365-2672.2004.02201.x

[24] Høiby N, Ciofu O, Bjarnsholt T (2010) Pseudomonas aeruginosa biofilms in cystic fibrosis. Future Microbiol 5: 1663–1674. 21133688 10.2217/fmb.10.125

[25] Coenye T (2010) Social interactions in the Burkholderia cepacia complex: biofilms and quorum sensing. Future Microbiol 5: 1087–1099. 20632807 10.2217/fmb.10.68

[26] Hassett DJ, Korfhagen TR, Irvin RT, Schurr MJ, Sauer K, et al (2010) Pseudomonas aeruginosa biofilm infections in cystic fibrosis: insights into pathogenic processes and treatment strategies. Expert Opin Ther Targets 14: 117–130. 20055712 10.1517/14728220903454988

[27] Huber B, Riedel K, Hentzer M, Heydorn A, Gotschlich A, et al (2001) The cep quorum-sensing system of Burkholderia cepacia H111 controls biofilm formation and swarming motility. Microbiology 147: 2517–2528. 11535791 10.1099/00221287-147-9-2517

[28] Tomlin KL, Coll OP, Ceri H (2001) Interspecies biofilms of Pseudomonas aeruginosa and Burkholderia cepacia. Can J Microbiol 47: 949–954. 11718549

[29] Lopes SP, Ceri H, Azevedo NF, Pereira MO (2012) Antibiotic resistance of mixed biofilms in cystic fibrosis: impact of emerging microorganisms on treatment of infection. Int J Antimicrob Agents In press. Available: http://dx.doi.org/10.1016/j.ijantimicag.2012.04.020. 10.1016/j.ijantimicag.2012.04.020 22770521

[30] Dalton T, Dowd SE, Wolcott RD, Sun Y, Watters C, et al (2011) An in vivo polymicrobial biofilm wound infection model to study interspecies interactions. PLoS ONE 6 11: e27317 doi:10.1371/journal.pone.0027317. 22076151 10.1371/journal.pone.0027317 PMC3208625

[31] Duan K, Dammel C, Stein J, Rabin H, Surette MG (2003) Modulation of Pseudomonas aeruginosa gene expression by host microflora through interspecies communication. Mol Microbiol 50: 1477–1491. 14651632 10.1046/j.1365-2958.2003.03803.x

[32] Bragonzi A, Paroni M, Nonis A, Cramer N, Montanari S, et al (2009) Pseudomonas aeruginosa microevolution during cystic fibrosis lung infection establishes clones with adapted virulence. Am J Respir Crit Care Med 180: 138–145. 19423715 10.1164/rccm.200812-1943OC

[33] Bragonzi A (2010) Murine models of acute and chronic lung infection with cystic fibrosis pathogens. Int J Med Microbiol 300: 584–593. 20951086 10.1016/j.ijmm.2010.08.012

[34] Stoicov C, Whary M, Rogers AB, Lee FS, Klucevsek K, et al (2004) Coinfection modulates inflammatory responses and clinical outcome of Helicobacter felis and Toxoplasma gondii infections. J Immunol 173: 3329–3336. 15322196 10.4049/jimmunol.173.5.3329

[35] Kosai K, Seki M, Tanaka A, Morinaga Y, Imamura Y, et al (2011) Increase of apoptosis in a murine model for severe pneumococcal pneumonia during influenza A virus infection. Jpn J Infect Dis 64: 451–457. 22116322

[36] Paroni M, Moalli F, Nebuloni M, Pasqualini F, Bonfield T, et al (2012) Response of CFTR-deficient mice to long-term Pseudomonas aeruginosa chronic infection and PTX3 therapeutic treatment. J Infect Dis in press, published on line: October 18,doi: 10.1093/infdis/jis636. 10.1093/infdis/jis636 PMC4055833 23087427

[37] Bragonzi A, Wiehlmann L, Klockgether J, Cramer N, Worlitzsch D, et al (2006) Sequence diversity of the mucABD locus in Pseudomonas aeruginosa isolates from patients with cystic fibrosis. Microbiology 152: 3261–3269. 17074897 10.1099/mic.0.29175-0

[38] Pirone L, Bragonzi A, Farcomeni A, Paroni M, Auriche C, et al (2008) Burkholderia cenocepacia strains isolated from cystic fibrosis patients are apparently more invasive and more virulent than rhizosphere strains. Environ Microbiol 10: 2773–2784. 18643926 10.1111/j.1462-2920.2008.01697.x

[39] Bevivino A, Pirone L, Pilkington R, Cifani N, Dalmastri C, et al (2012) Interaction of environmental Burkholderia cenocepacia strains with cystic fibrosis and non-cystic fibrosis bronchial epithelial cells in vitro. Microbiology 158: 1325–1333. 22322958 10.1099/mic.0.056986-0

[40] Hutchison ML, Poxton IR, Govan JR (1998) Burkholderia cepacia produces a hemolysin that is capable of inducing apoptosis and degranulation of mammalian phagocytes. Infect Immun 66: 2033–2039. 9573086 10.1128/iai.66.5.2033-2039.1998 PMC108160

[41] Macho AP, Zumaquero A, Ortiz-Martin I, Beuzon CR (2007) Competitive index in mixed infections: a sensitive and accurate assay for the genetic analysis of Pseudomonas syringae-plant interactions. Mol Plant Pathol 8: 437–450. 20507512 10.1111/j.1364-3703.2007.00404.x

[42] Burmølle M, Webb JS, Rao D, Hansen LH, Sørensen SJ, et al (2006) Enhanced biofilm formation and increased resistance to antimicrobial agents and bacterial invasion are caused by synergistic interactions in multispecies biofilms. Appl Environ Microbiol 72: 3916–3923. 16751497 10.1128/AEM.03022-05 PMC1489630

[43] Ryan RP, Fouhy Y, Garcia BF, Watt SA, Niehaus K, et al (2008) Interspecies signalling via the Stenotrophomonas maltophilia diffusible signal factor influences biofilm formation and polymyxin tolerance in Pseudomonas aeruginosa. Mol Microbiol 68: 75–86. 18312265 10.1111/j.1365-2958.2008.06132.x

[44] Ryan RP, Lucey J, O'Donovan K, McCarthy Y, Yang L, et al (2009) HD-GYP domain proteins regulate biofilm formation and virulence in Pseudomonas aeruginosa. Environ Microbiol 11: 1126–1136. 19170727 10.1111/j.1462-2920.2008.01842.x

[45] van Heeckeren AM, Schluchter MD, Drumm ML, Davis PB (2004) Role of Cftr genotype in the response to chronic Pseudomonas aeruginosa lung infection in mice. Am J Physiol Lung Cell Mol Physiol 287: L944–952. 15246977 10.1152/ajplung.00387.2003

[46] Bragonzi A, Worlitzsch D, Pier GB, Timpert P, Ulrich M, et al (2005) Nonmucoid Pseudomonas aeruginosa expresses alginate in the lungs of patients with cystic fibrosis and in a mouse model. J Infect Dis 192: 410–419. 15995954 10.1086/431516 PMC1317300

