# Supplementary figures and images for: Modelling Co-Infection of the Cystic Fibrosis Lung by Pseudomonas aeruginosa and Burkholderia cenocepacia Reveals Influences on Biofilm Formation and Host Response
Source: PLoS One. 2012 Dec 21;7(12):e52330. doi: 10.1371/journal.pone.0052330 (PMC3528780; doi:10.1371/journal.pone.0052330)

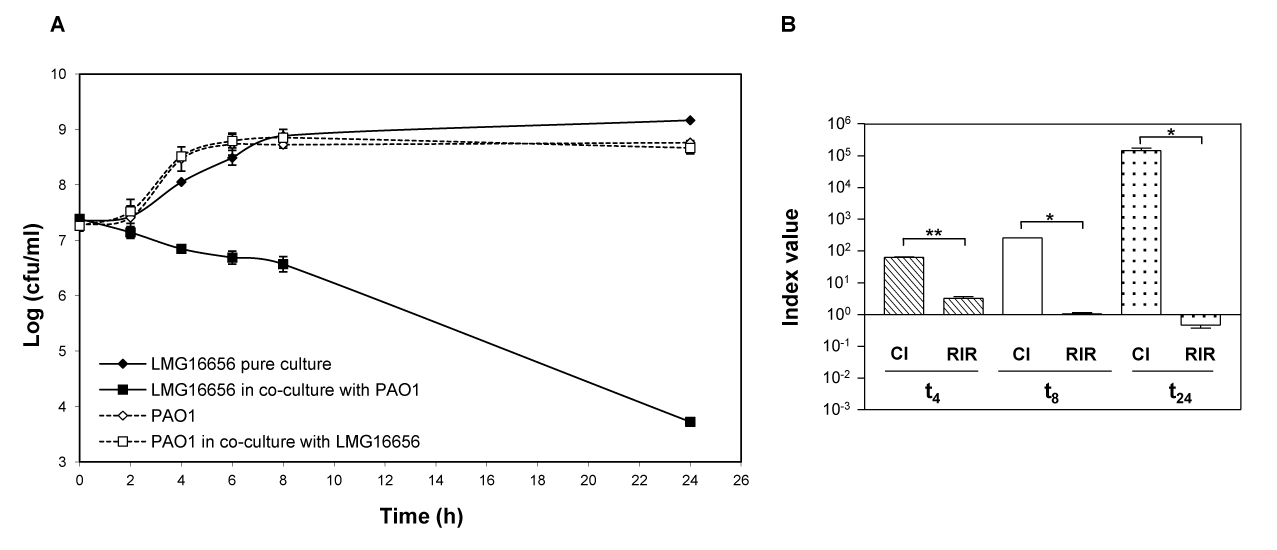

Supplement: Figure S1 — Single and dual species batch growth curves of laboratory P. aeruginosa PAO1 and clinical B. cenocepacia LMG16656 strains and the competitive index (CI) and relative increase ratio (RIR) values. (A) The two species were individually cultured or co-cultured at a 1∶1 ratio and grown for 24 h in NB medium at 37°C with vigorous aeration. Colony-forming unit counts (CFU) was determined at 0, 2, 4, 6, 8 and 24 h of bacterial growth. The results are the mean of Log (cfu/mL) values of three separate assays. Key: (A) Growth of laboratory P. aeruginosa PAO1 and B. cenocepacia LMG16656 strains in single and dual cultures; (B) CI and RIR generated from single and dual cultures of laboratory P. aeruginosa PAO1 and B. cenocepacia LMG16656 strains. CI and RIR were calculated as described in Materials and Methods . Each value represents the mean of RIR and CI values from three separate assays, and the bars indicate standard deviations. * = P<0.05, ** = P<0.01 in the Student's t test. (TIF) [file pone.0052330.s001.tif]

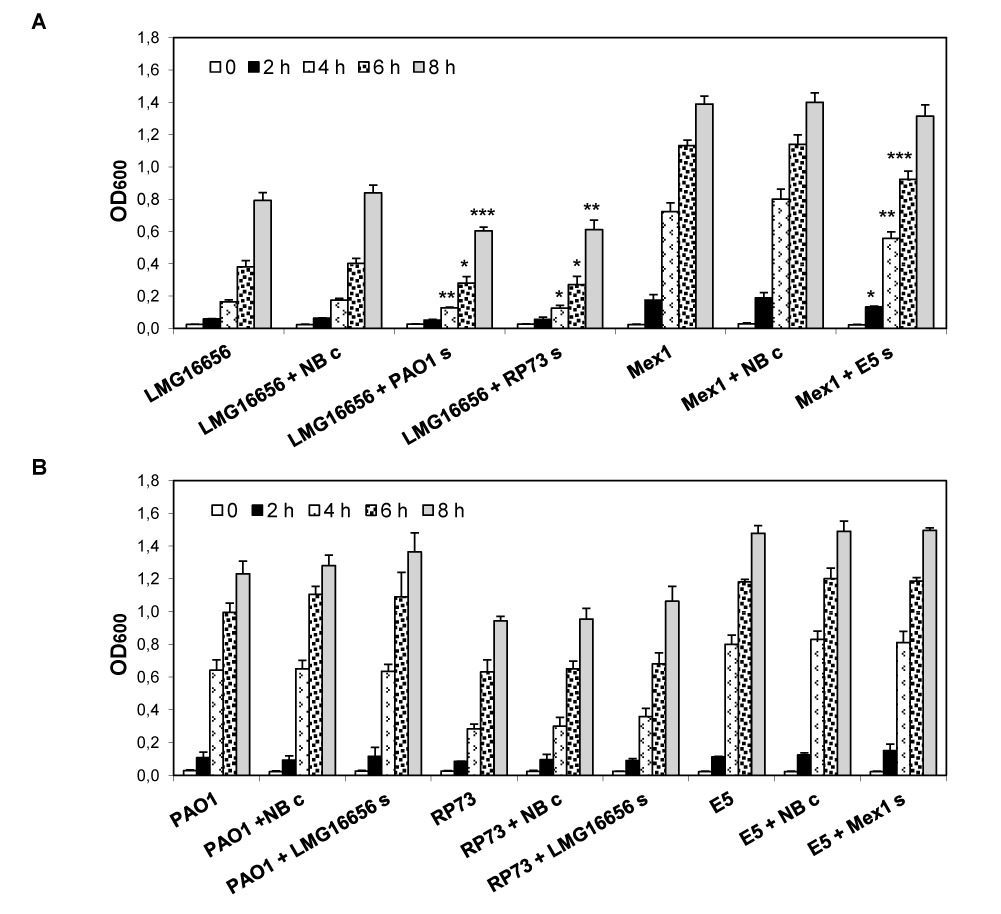

Supplement: Figure S2 — Effect of the supernatants on planktonic bacterial growth. (A) Effect of the supernatant of P. aeruginosa cultures on growth of planktonic cultures of B. cenocepacia. (B) Effect of the supernatant of B. cenocepacia cultures on growth planktonic cultures of P. aeruginosa. The two species were grown at 37°C with vigorous aeration in NB medium supplemented with sterile concentrated supernatant of the second organism at a final concentration of 1×. As controls, pure cultures were grown in NB medium alone and in NB medium supplemented with concentrated NB medium to a final concentration of 1×. OD600 was measured at 0, 2, 4, 6 and 8 h of bacterial growth. The means ± standard deviations for at least three separate assays are illustrated. * = P<0.05, ** = P<0.01, *** = P<0.001 in the Student's t test with respect to the pure cultures grown in NB medium; s = supernatant; NBc = concentrated Nutrient Broth medium. (TIF) [file pone.0052330.s002.tif]

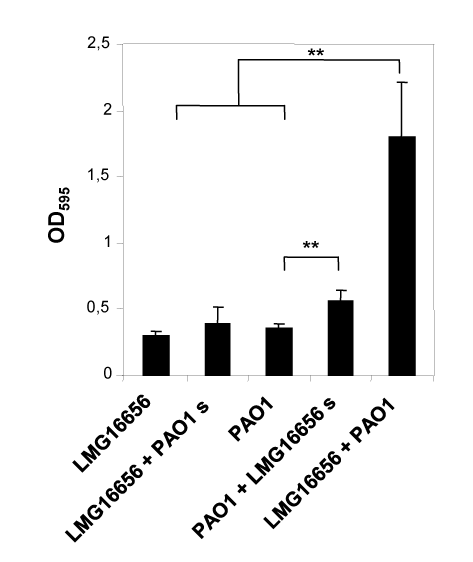

Supplement: Figure S3 — Biofilm formation by laboratory P. aeruginosa PAO1 and clinical B. cenocepacia LMG16656 strains in single and dual cultures. Bacteria were grown overnight in 96-well polyvinyl chloride flat-bottomed microtiter plates in NB medium at 37°C either individually cultured or cocultured at a 1∶1 ratio or when individually cultured supplemented with sterile concentrated supernatant of the second organism at a final concentration of 1×. Biofilm biomass was quantified by staining with crystal violet and absorbance measurements at OD 595. The values are means of three separate assays, and the bars indicate standard deviation. * = P<0.05, ** = P<0.01 in Student's t test. s = supernatant. (TIF) [file pone.0052330.s003.tif]

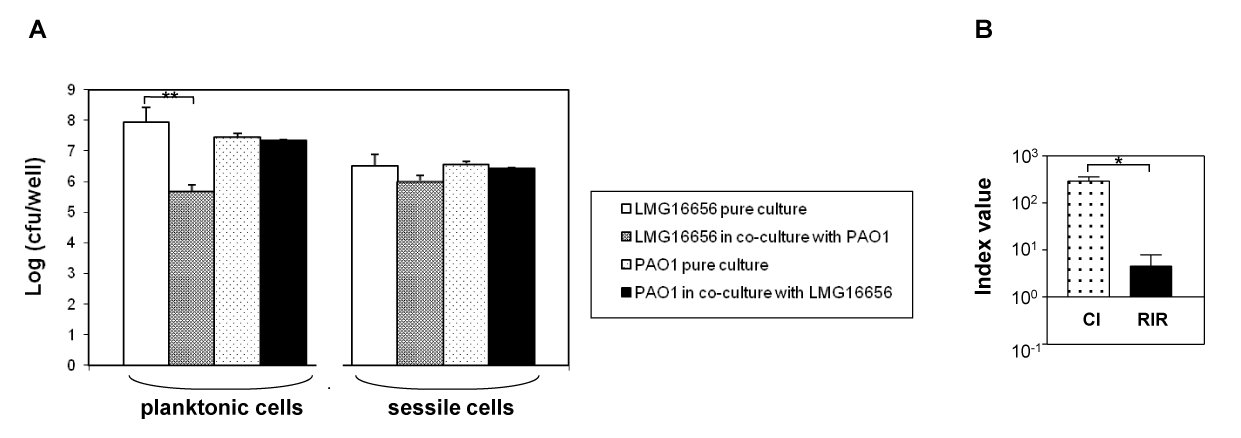

Supplement: Figure S4 — Laboratory P. aeruginosa PAO1 and clinical B. cenocepacia LMG16656 planktonic and sessile cells in single and dual cultures and the competitive index (CI) and relative increase ratio (RIR) values. Bacteria were grown overnight in 96-well polyvinyl chloride flat-bottomed microtiter plates in NB medium at 37°C either individually cultured or cocultured at a 1∶1 ratio. CFU counts were determined at 24 h of bacterial growth in both planktonic and sessile fraction. Key: (A) Planktonic (left) and sessile (right) cells of laboratory P. aeruginosa PAO1 and clinical B. cenocepacia LMG16656 in single and dual cultures; (B) CI and RIR mean values of planktonic growth of laboratory P. aeruginosa PAO1 versus clinical B. cenocepacia LMG16656. Each value represents the mean of RIR and CI values from three separate assays, and the bars indicate standard deviations. * = P<0.05, ** = P<0.01 in Student's t test. (TIF) [file pone.0052330.s004.tif]

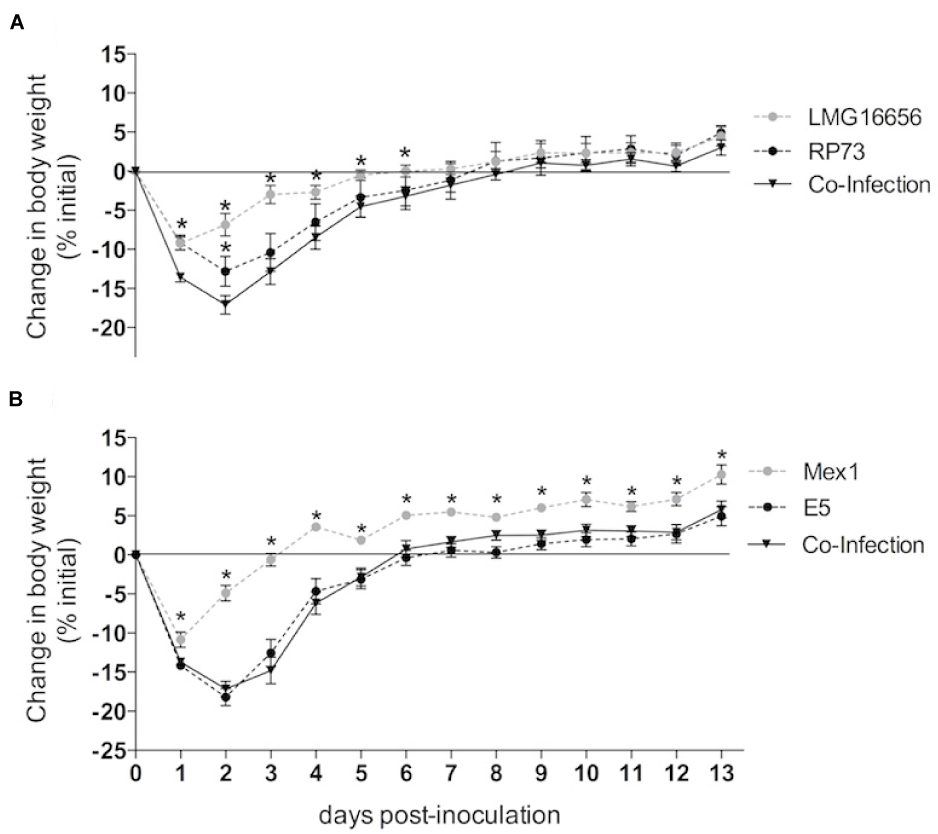

Supplement: Figure S5 — Weight change after infection with P. aeruginosa and B. cenocepacia alone or in co-infection. C57Bl/6NCrlBR mice were infected with P. aeruginosa, B. cenocepacia strains alone or in combination and monitored for weight change. Values are the mean daily weight gain over 13-day infection. Key: (A) Co-infection with clinical strains: mice co-infected with both pathogens lost significantly more weight than mice infected with B. cenocepacia LMG16656 alone from days 1 to 6; (B) Co-infection with environmental strains: mice infected with P. aeruginosa E5 alone or in coinfection lost significantly more weight than mice infected with B. cenocepacia Mex1 alone. (TIF) [file pone.0052330.s005.tif]
