# Supplementary material for: Modelling Co-Infection of the Cystic Fibrosis Lung by Pseudomonas aeruginosa and Burkholderia cenocepacia Reveals Influences on Biofilm Formation and Host Response
Source: PLoS One. 2012 Dec 21;7(12):e52330. doi: 10.1371/journal.pone.0052330 (PMC3528780; doi:10.1371/journal.pone.0052330)
Supplement: Table S1 — Colonization of murine lung with clinical and environmental P. aeruginosa and B. cenocepacia strains. (DOCX) [file pone.0052330.s006.docx]

**Table S1. Colonization of murine lung with clinical and environmental *P. aeruginosa* and *B. cenocepacia* strains.**

| **Mouse strain** | **Bacterial strain** | **No of mice^a^** | **Mortality %**  **(No of dead/total mice) ^b^** | **Chronic infection %**  **(No of infected/**  **surviving mice)^c^** | | **% of co-infected mice by single species^d^** | **Total CFU/lung of surviving mice (strain)** |
| --- | --- | --- | --- | --- | --- | --- | --- |
| **C57Bl/6NCrlBR** | | | | | | | |
|  | **RP73** | 17 | 5.88 (1/17) | 68.75 (11/16) |  | | 5.2x10^4^ |
|  | **Co-infection** | 22 | 0 (0/22) | 77.27 (17/22) | 100 (17/17) (RP73)  0 (0/17) (LMG16656) | | 4.2x10^4^ (RP73)  0 (LMG16656) |
|  | **LMG16656** | 15 | 0 (0/15) | 73.33 (11/15) |  | | 7.9x10^3^ |
|  | **E5** | 20 | 24 (6/25) | 36.84 (7/19) |  | | 2.6x10^4^ |
|  | **Co-infection** | 25 | 15 (3/20) | 58.82 (10/17) | 100 (10/10) (E5)  0 (0/10) (Mex1) | | 5.4x10^3^ (E5)  0 (Mex1) |
|  | **Mex1** | 16 | 0 (0/16) | 43.75 (7/16) |  | | 4.8x10^4^ |
| **B6.129P2-*Cftr^tm1UNC^TgN(FABPCFTR) Cftr*^+/+^** | | | | | | | |
|  | **RP73** | 11 | 18.18 (2/11) | 88.88 (8/9) |  | | 1.5x10^4^ |
|  | **Co-infection** | 19 | 26.31 (5/19) ^#^ | 64.28 (9/14) | 100 (9/9) (RP73)  11.11 (1/9) (LMG16656) | | 7.9x10^4^ (RP73) 1.2x10^7^(LMG16656) |
|  | **LMG16656** | 14 | 14.28 (2/14) | 100 (12/12) |  | | 2.2x10^5^ |
| **B6.129P2-*Cftr^tm1UNC^TgN(FABPCFTR)Cftr^S489X/S489X^*** | | | | | | | |
|  | **RP73** | 10 | 20 (2/10) | 75 (6/8) |  | | 2.4x10^5^ |
|  | **Co-infection** | 15 | 26.66 (4/15) ^#^ | 81.81 (9/11) | 100 (9/9) (RP73)  11.11 (1/9) (LMG16656) | | 4x10^4^ (RP73) 8.4x10^4^(LMG16656) |
|  | **LMG16656** | 11 | 18.8 (2/11) | 88.88 (8/9) |  | | 6.65x10^4^ |

^a^ Pooled mice, analysed in two to three independent experiments.

^b^ Mortality 14 days after challenge expressed as median values. ^#^Significant difference of C57Bl/6NCrlBR *vs* B6.129P2-*Cftr^tm1UNC^TgN(FABPCFTR) Cftr*^+/+^ and B6.129P2-*Cftr^tm1UNC^TgN(FABPCFTR)Cftr^S489X/S489X^* (^#^ *P*<0.05). Chi-square test (two-tailed).

^c^ Infected mice, surviving 14 days after challenge. Data represent median values of infected mice, surviving 14 days after challenge.

^d^ For co-infection experiments data indicate the percentage of infected mice by *P. aeruginosa* or *B. cenocepacia*. In parenthesis, the number of infected mice by single species or co-infected mice.
